# Identification, characterization and expression analysis of calmodulin and calmodulin-like proteins in *Solanum pennellii*

**DOI:** 10.1038/s41598-020-64178-y

**Published:** 2020-05-04

**Authors:** Jinyan Shi, Xiangge Du

**Affiliations:** College of Plant Protection, China Agricultural University, Beijing, China

**Keywords:** Plant physiology, Plant stress responses

## Abstract

In plants, the calmodulin (CaM) proteins is an important calcium-binding protein, which play a crucial role in both regulating plant growth and development, as well as in the resistance mechanisms to various biotic and abiotic stresses. However, there is limited knowledge available on the CaM family functions in *Solanum pennellii*, a wild tomato species utilized as a genetic resource for cultivated tomatoes. In this study, 6 *CaM* (*SpCaM*) and 45 *CaM*-like (*SpCML*) genes from *Solanum pennellii* were selected for bioinformatics analysis to obtain insights into their phylogenetic relationships, gene structures, conserved motifs, chromosomal locations, and promoters. The results showed that the 6 SpCaM proteins contained 4 EF-hand domains each, and the 45 SpCML proteins had 2-4 EF-hand domains. The 51 *CaM* and *CaM*-like genes contained different intron/exon patterns and they were unevenly distributed across the 12 chromosomes of *S. pennellii*. The results of the analysis of the conserved motifs and promoter cis-regulatory elements also indicated that these proteins were involved in the responses to biotic and abiotic stresses. qRT-PCR analysis indicated that the *SpCaM* and *SpCML* genes had broad expression patterns in abiotic stress conditions and with hormone treatments, in different tissues. The findings of this study will be important for further investigations of the calcium signal transduction mechanisms under stress conditions and lay a theoretical foundation for further exploration of the molecular mechanisms of plant resistance.

## Introduction

Plants are invariably subjected to stressful environmental conditions and pathogenic attacks from various bacteria, fungi, and viruses throughout their lives. To survive these biotic and abiotic stresses, plants have evolved adaptive molecular mechanisms, many of which involve calcium as a second messenger in cellular stress signal transductions^1–4^. When threatened, the rapid increase of Ca^2+^ concentrations in the cytoplasm causes calcium transients and calcium oscillations, which are the initial responses to the stimulus^5^, and lead to calcium being bound to sensor responders or sensor relays^2^. The sensor responders can combine calcium to generate signals directly and have functions as both sensors and effectors, which mainly include calcium-dependent protein kinase (CDPK) proteins^6^. Sensor relays bound by Ca^2+^, however, need to interact with the target proteins to produce their signals, and they are mainly protein phosphatases, such as calmodulin (CaM), CaM-like (CML), and calcineurin B-like (CBL) proteins^7^.

CaM, is an essential calcium-binding protein, that has been identified in plants, several protozoa, and animals^8^. In plants, the typical CaM structure contains approximately 150 amino acid residues, and the structures of the different CaM proteins are highly conserved. CaM carries 4 EF-hand type calcium-binding domains that are 12 amino acid residues long each. Each of the 4 EF-hand domains binds to a Ca^2+^, and this binding changes the conformation of the CaM, thereby activating it to perform signal transductions. Another class of proteins, called CML, are structurally similar to the CaM and contain 1 to 4 EF-hand domains. Unlike the CaM proteins, some of CML EF-hand domains are not Ca^2+^ binding regions^9–11^. The CaM and CML family proteins have been identified in numerous plants. By means of genome-wide analysis, 6 CaM and 50 CML have been identified in *Arabidopsis*^12^. Furthermore, the genome of *Oryza sativa* was found to encode 5 CaM and 32 CML^13^, *Solanum lycopersicum* was found to 24 CML^14^, and soybean had 6 CaM and 144 CML^15^. They have also been identified in other cash crops, such as *Vitis vinifera* (3 CaM and 62 CML)^4^, *Gossypium raimondii* (6 CaM and 30 CML)^11^, *Brassica rapa* L. (79 CML)^16^, and *Nicotiana benthamiana* (7 CaM and 55 CML)^8^. However, currently, there is little biological information available on the CaM and CML families in *Solanum pennellii*.

The *CaM* and *CML* genes are widely distributed in plant cells, and there are different levels present in the tissues, and protoplasts^12,14,16,17^. Increasing evidence shows that *CaM* and *CML* genes play a vital role in plant growth and development, cell metabolism, and disease resistance^18–22^. During pollen germination and tube elongation in Arabidopsis, K + influx dependent on Ca^2+^ is regulated by AtCML25^23^; AtCML39 is significantly expressed in the process of light signal transduction to promoting seedling growth^24^. In trichome, kinesin-interacting Ca^2+^-binding protein (KIC) is a novel Ca^2+^ binding protein with an EF-hand motif, and modulates microtubule motor protein in response to changes in cytosolic Ca^2+^ and negatively regulates trichome stalk length and branching^25^. AtCML42 interacts with KIC to transmit the calcium signal downstream, which regulates the cell branch of the trichome^26^. The FLOWERING LOCUS C (FLC) is a negative regulator of plant flowering^27^. AtCML23 and AtCML24 proteins reduce the level of NO by transmitting calcium signals, which result in inhibition of the *FLOWERING LOCUS C* (FLC) gene expression, thus affecting the autonomic regulatory pathway of the transition to flowering^28^.

In addition, the functional roles in the various adversity stress are also revealed. Overexpression of MtCML40 causes the down-regulation of MtHKT1 (Na+ transport proteins 1) and led to greater accumulation of Na+ in shoots; thus rendering the transgenic *M. truncatula* seedlings more sensitive to salt stress^29^. In eukaryotes, MAPK (mitogen-activated protein kinase) phosphatase (MKPs) are negative regulators of MAPKs. The wheat MKP (TMKP) contains a CaM binding domain and binds to CaM in a Ca^(2+)^-dependent manner^30^. The CaM/Ca^2+^ complex inhibits the catalytic activity of TMKP, but this activity was enhanced by the complex formations between CaM/Ca^2+^ and Mn^2+^. The dual regulation was mediated via the interactions between CaM/Ca^2+^ and the TMKP1 C-terminal CaM binding domain^30^. In tomatoes, CML43 was involved in the immune response to pathogens^31^. The overexpression of *Arabidopsis* CML8, improved resistance to pathogenic bacteria^32^.

The stress-tolerant wild tomato species *Solanum pennelllii* is a major genetic resource for the improvement of cultivated tomatoes^33^. For example, resistance genes have been discovered in *Solanum pennelllii* for powdery mildew^34^, and whitefly *Bemisia argentifolii*^35^, as well as genes associated with drought^36^ and salt tolerance^37^. In this study, we have selected 6 *CaM* and 45 *CML* genes from the *Solanum pennellii* genome and have performed bioinformatics analysis that included phylogenetic analysis, chromosomal localization, protein physicochemical parameter predictions, exon-intron structure analysis, and conserved motifs and cis-acting elements of the promoter region analysis, which provided basic information for the discovery of stress-response related genes in wild tomatoes and candidate genes for developing tomato stress-tolerant cultivars.

## Results

### Biochemical characteristics of the SpCaM and SpCML proteins

In previous studies, the amino acid sequences of the CaM and CML proteins in *Arabidopsis* and rice have been reported. In this study, 51 non-redundant sequences were identified in the *Solanum pennellii* genome, including 6 SpCaM and 45 SpCML^33^. All of the SpCaM and SpCML proteins were named according to their amino acid identity percentage with true canonical CaM7 (AtCaM7)^12,13^. Then, the biochemical characteristics of these proteins were predicted using the ExPASy proteomics server^38^ and Wolf PSORT program^39^ (Table 1). These SpCaM proteins shared more than 90% sequence similarity with AtCaM7. The number of amino acids (aa), molecular weight, isoelectric point (pi), and percentage of methionine in all the SpCaM proteins, except for SpCaM3, was 149, 16.8 kDa, 4.1, and 6.0%, respectively. The number of amino acids in the SpCML proteins varied from 129 to 282, except for SpCML43, which contained 340 amino acids. The molecular weights of the SpCML proteins ranged from 14.7 to 36.1 kDa, and their pi and percentage methionine ranged from 3.9 to 9.5 and 0.9 to 8.6%, respectively. Except for the absence of cysteine in SpCML5, SpCML9, SpCML12, and SpCML19, the rest of the SpCaM and SpCML proteins contained both cysteine and lysine. All SpCML and SpCaM proteins lacked the N-myristoylation sites, except for SpCML5, SpCML8, and SpCML36. SpCaM1-SpCaM6 possessed a standard structure characterized by 4 EF-hand type calcium-binding regions. The number of SpCML EF-hand domains varied from 1 to 4. The predicted results for the protein subcellular localizations of SpCaM and SpCML are listed in Table 1.

**Table 1:** Characteristics and names of the SpCaM and SpCML proteins identified in the *Solanum pennellii* genome.

Gene name	Gene ID	aa	% of amino acids identity to ATCaM7	EF-Hand No.	calcium-bindingregion No.	Mol Wt(kDa)	pi	Percentage methionine	Presence of cysteine	Presence of lysine	Potential myristoylation site	Sub-cell localization^a^
SpCaM1	107002231	149	100.00	4	4	16.85	4.11	6.00%	+	+		nucl: 5, mito: 3, extr: 3, cyto: 2, chlo: 1
SpCaM2	107001961	149	99.33	4	4	16.83	4.10	6.00%	+	+		nucl: 5, mito: 3, extr: 3, cyto: 2, chlo: 1
SpCaM3	107004451	180	99.33	4	4	20.51	4.61	6.70%	+	+		nucl: 5.5, cyto_nucl: 4.5, chlo: 4, cyto: 2.5, extr: 2
SpCaM4	107007179	149	99.33	4	4	16.83	4.10	6.00%	+	+		nucl: 5, mito: 3, extr: 3, cyto: 2, chlo: 1
SpCaM5	107008025	149	98.66	4	4	16.85	4.11	6.00%	+	+		nucl: 5, mito: 3, extr: 3, cyto: 2, chlo: 1
SpCaM6	107015299	149	91.95	4	4	16.93	4.15	6.00%	+	+		nucl: 7, cyto: 2, plas: 2, mito: 1, extr: 1, cysk: 1
SpCML1	107022582	149	78.23	4	4	16.95	3.96	6.00%	+	+		chlo: 4, cyto: 4, extr: 4, nucl: 1, cysk: 1
SpCML2	107013078	150	66.67	3	2	17.44	4.34	5.30%	+	+		cyto: 8.5, cyto_nucl: 7, nucl: 2.5, mito: 1, extr: 1, cysk_plas: 1
SpCML3	107029662	147	65.31	4	3	16.95	4.08	4.80%	+	+		cyto_nucl: 6, cyto: 5, chlo: 3, nucl: 3, extr: 3
SpCML4	107016234	191	50.73	4	4	21.09	4.40	4.70%	+	+		nucl: 11, chlo: 2, cyto: 1
SpCML5	107015376	147	50.34	3	1	16.53	4.90	3.40%		+	+	cyto: 7, plas: 3, chlo: 1, nucl: 1, extr: 1, golg: 1
SpCML6	107028473	172	50.00	4	4	19.15	4.33	5.80%	+	+		nucl: 5, cyto: 4, extr: 2, chlo: 1, mito: 1, golg_plas: 1
SpCML7	107014545	163	48.65	4	4	18.01	4.35	3.10%	+	+		nucl: 5.5, nucl_plas: 5.5, plas: 4.5, cyto: 2, chlo: 1, mito: 1
SpCML8	107004453	147	46.98	3	1	16.56	4.89	4.10%	+	+	+	plas: 4, nucl: 3, cyto: 3, chlo: 1, extr: 1, pero: 1, golg: 1
SpCML9	107026854	163	45.89	4	4	18.03	4.48	2.50%		+		nucl_plas: 6.5, nucl: 6, plas: 5, chlo: 2, mito: 1
SpCML10	107011246	156	45.46	4	4	17.08	4.93	4.50%	+	+		chlo: 10, nucl: 4
SpCML11	107010502	198	43.08	2	2	22.76	4.63	3.00%	+	+		cyto: 3, E.R.: 3, mito: 2, vacu: 2, chlo: 1, plas: 1, extr: 1, golg: 1
SpCML12	107027412	161	41.10	4	4	17.68	4.24	6.20%		+		cyto: 8.5, cyto_nucl: 8, nucl: 4.5, chlo: 1
SpCML13	107013815	202	40.91	2	2	23.26	4.23	4.00%	+	+		vacu: 10, plas: 2, extr: 1, golg: 1
SpCML14	107018599	150	40.88	4	4	17.00	4.33	6.70%	+	+		cyto: 8, chlo: 2, nucl: 2, mito: 1, cysk: 1
SpCML15	107021713	282	40.88	4	4	32.09	5.34	4.30%	+	+		chlo: 11, mito: 2, nucl: 1
SpCML16	107004268	151	40.58	4	3	17.29	4.10	7.90%	+	+		cyto: 4, nucl: 3, mito: 3, chlo: 2, extr: 2
SpCML17	107009322	145	40.58	4	4	16.19	4.92	5.50%	+	+		nucl: 7, cyto: 5, extr: 2
SpCML18	107030245	191	39.86	4	3	21.98	4.45	6.30%	+	+		cyto: 4.5, chlo: 4, cyto_nucl: 3, extr: 2, mito: 1, plas: 1, pero: 1
SpCML19	107014001	213	39.60	4	4	23.88	5.14	4.20%		+		chlo: 5, vacu: 2, E.R.: 2, nucl: 1, cyto: 1, mito: 1, pero: 1, golg: 1
SpCML20	107028975	192	39.58	4	4	21.21	4.75	4.70%	+	+		nucl: 9, chlo: 2.5, cyto: 2, chlo_mito: 2
SpCML21	107015792	167	36.24	4	4	18.33	4.28	5.40%	+	+		cyto: 6, mito: 4, nucl: 2, chlo: 1, extr: 1
SpCML22	107005687	238	35.95	4	4	27.01	4.82	4.20%	+	+		chlo: 8, extr: 3, nucl: 1, cyto: 1, vacu: 1
SpCML23	107014219	141	35.71	4	3	16.09	4.43	5.70%	+	+		cyto: 10, nucl: 2, cysk: 1, golg: 1
SpCML24	107009143	193	35.46	4	4	21.67	7.69	7.30%	+	+		chlo: 7, mito: 4, nucl: 2, pero: 1
SpCML25	107023142	185	35.42	3	2	21.25	4.40	2.20%	+	+		cyto: 4, chlo: 2, plas: 2, extr: 2, vacu: 2, mito: 1, E.R.: 1
SpCML26	107012744	193	34.56	4	3	22.34	5.33	3.60%	+	+		mito: 8, chlo: 3, cyto_nucl: 2, nucl: 1.5, cyto: 1.5
SpCML27	107005592	208	34.29	4	4	23.72	9.01	3.80%	+	+		chlo: 7, plas: 3, nucl: 2, cyto: 1, golg: 1
SpCML28	107022660	186	33.09	4	3	20.66	4.70	6.50%	+	+		nucl: 11, chlo: 2, extr: 1
SpCML29	107005005	197	32.41	4	3	22.30	4.94	8.60%	+	+		extr: 6, nucl: 5, cyto: 1, vacu: 1, golg: 1
SpCML30	107015719	214	32.03	4	2	23.39	4.44	2.80%	+	+		nucl: 4, mito: 4, chlo: 3, extr: 2, cyto: 1
SpCML31	107010362	230	31.54	3	2	26.69	5.05	3.00%	+	+		chlo: 4, extr: 3, mito: 2, vacu: 2, nucl: 1, E.R.: 1, golg: 1
SpCML32	107010462	141	30.94	4	3	16.30	4.51	5.70%	+	+		cyto: 7, nucl: 2, mito: 2, extr: 2, plas: 1
SpCML33	107004057	178	30.66	4	3	20.11	4.89	7.90%	+	+		nucl: 7, cyto: 4, mito: 2, extr: 1
SpCML34	107005004	198	30.66	4	3	21.87	4.79	5.60%	+	+		nucl: 8, chlo: 2, cyto: 1, mito: 1, extr: 1, golg: 1
SpCML35	107025899	221	26.36	4	4	25.03	4.54	3.20%	+	+		extr: 7, chlo: 3, vacu: 3, cyto: 1
SpCML36	107032262	230	25.93	4	2	26.26	4.47	3.50%	+	+	+	cyto: 4, chlo: 3, plas: 3, E.R.: 2, mito: 1, vacu: 1
SpCML37	107011884	159	23.78	3	3	17.31	4.38	4.40%	+	+		cyto: 14
SpCML38	107016285	179	17.71	2	2	20.18	5.04	2.80%	+	+		nucl: 7, cyto: 3, plas: 2, chlo: 1, extr: 1
SpCML39	107031954	209	15.96	4	3	23.43	4.95	3.30%	+	+		mito: 7, nucl: 4.5, cyto_nucl: 3, chlo: 2
SpCML40	107017051	129	15.91	4	2	14.79	9.46	2.30%	+	+		mito: 6, cyto: 4.5, cyto_E.R.: 3, nucl: 2, chlo: 1
SpCML41	107017137	159	14.85	3	2	18.07	4.49	2.60%	+	+		cyto_nucl: 7.5, cyto: 7, nucl: 4, extr: 2, cysk: 1
SpCML42	107017114	158	14.53	3	2	18.11	4.25	4.40%	+	+		nucl: 4, cyto: 4, chlo: 3, plas: 2, extr: 1
SpCML43	107002660	340	13.87	2	2	36.05	6.30	0.90%	+	+		nucl: 12, cyto: 1, plas: 1
SpCML44	107029131	211	13.50	4	1	24.18	5.25	0.90%	+	+		chlo: 6, cyto: 2, mito: 2, plas: 2, extr: 1, E.R.: 1
SpCML45	107003124	250	13.40	2	2	28.25	6.43	2.40%	+	+		chlo: 6, nucl: 4, extr: 2, cyto: 1, mito: 1

^a^Nucl, nuclear; ER, endoplasmic reticulum; Mito, mitochondria; Extr, extracellular; Cyto, cytosol; Chlo, chloroplast; Plas, plasma membrane; Cysk, cytoskeleton; Golg, Golgi apparatus; Pero, peroxisomes; Vacu, vacuolar membrane

### Phylogenetic analysis of SpCaM and SpCML families

**Figure 1:**
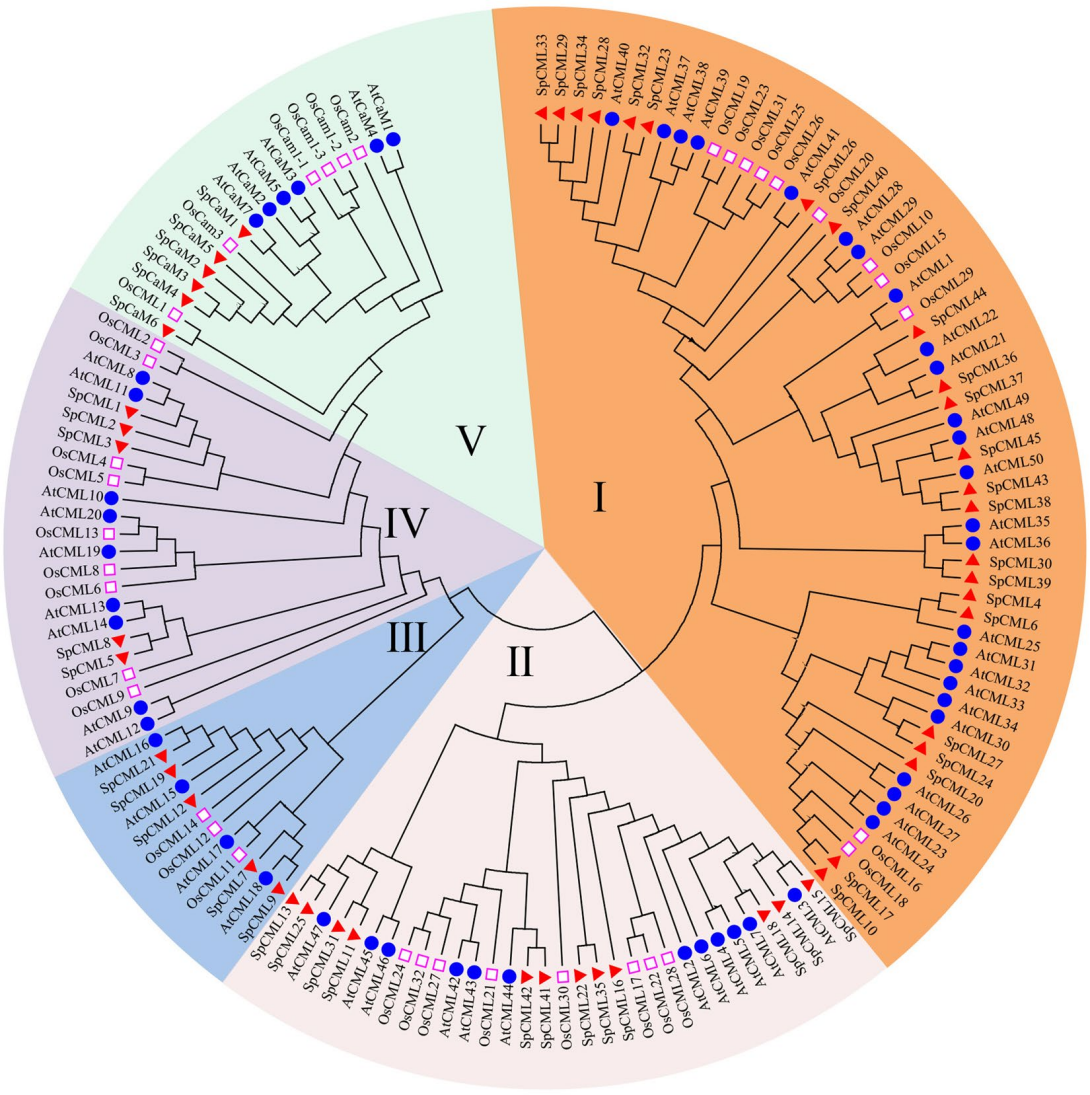
Phylogenetic relationship among CaM and CML proteins from *Solanum pennellii*, rice, and *Arabidopsis*. I-V indicated different gene groups. To identify the plant species origin of each CaM and CML, a species acronym was included before the protein name: eg. SpCaM indicated CaM from *Solanum pennellii*, AtCaM from *Arabidopsis* and OsCaM from rice. The red triangle, blue dots and pink border square indicated proteins from *Solanum pennellii*, *Arabidopsis* and rice, respectively.

The phylogenetic relationships between the CaM and CML family members of the *Solanum pennellii*, *Arabidopsis*, and rice were analyzed using the neighbor-joining method of MEGA6.0^40^. The CaM and CML of the three species were divided into five groups (Fig. 1). The 6, 6, and 5 CaM proteins of the *Solanum pennellii, Arabidopsis*, and rice, respectively, were individually classified into group V, which was closest to group IV, which was made up of 5 SpCML, 9 AtCML, and 9 OsCML. Only one CML (OsCML-1) existed in group V. In the phylogenetic tree, groups I and III were the largest and the smallest with 59 and 12 CML proteins, respectively. Group I consisted 23 SpCML, 25 AtCML, and 11 OsCML, while group III consisted 5 SpCML, 4 AtCML, and 3 OsCML. Group II consisted 12 SpCML, 12 AtCML, and 8 OsCML.

The dendrogram showed that the proteins of the *Solanum pennellii* were generally closer to the proteins of *Arabidopsis* than those of rice, suggesting the phylogenetic relationship between *Solanum pennellii* and *Arabidopsis* is relatively closer.

### Genomic distribution of *SpCaM* and *SpCML* genes

**Figure 2:**
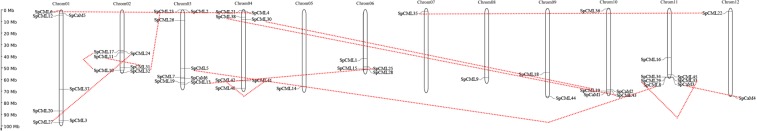
Locations of *SpCaM* and *SpCML* genes on chromosomes. The scale is in megabase (Mb). Paralogous gene are connected by broken red lines.

To determine the distributions of the 6 *SpCaM* and 45 *SpCML* on the chromosomes, their physical locations were searched using the NCBI database and were mapped to 12 chromosomes using online MapGene2Chrom program^41^. As can be seen in Fig. 2, the 6 *SpCaM* and 45 *SpCML* were unevenly distributed across the 12 chromosomes. Chromosomes 5, 7, and 8 contained only one gene (*SpCML*), while chromosome 3 contained the most genes (7 *SpCML* and 1 *SpCaM*). Chromosomes 1, 4, and 11 all contained seven genes. The respective number of genes located on chromosomes 2, 6, 9, 10, and 12 were 6, 4, 2, 5, and 2, respectively. The 6 *SpCaM* genes were distributed on five chromosomes (chromosomes 1, 3, 10, 11, and 12, which contained 1, 1, 2, 1, and 1 gene, respectively. There was only one pair of *SpCaM* paralogous genes and two *SpCaM* genes (*SpCaM3* and *SpCaM4*) on chromosomes 11 and 12, respectively. There were two pairs of paralogous genes (*SpCML10/SpCML17* and *SpCML11/SpCML31*) on chromosome 2, while the other two pairs of paralogous genes (*SpCML41/SpCML42* and *SpCML29/SpCML33*) existed on chromosomes 4 and 11, respectively. The other *SpCML* paralogous genes (*SpCML4/SpCML6, SpCML5/SpCML8, SpCML13/SpCML25, SpCML22/SpCML35, SpCML23/SpCML32, SpCML24/SpCML27, SpCML30/SpCML39*, and *SpCML38/SpCML43*) appeared on different chromosomes.

### Genetic structure analysis of the *SpCaM* and *SpCML* genes

**Figure 3:**
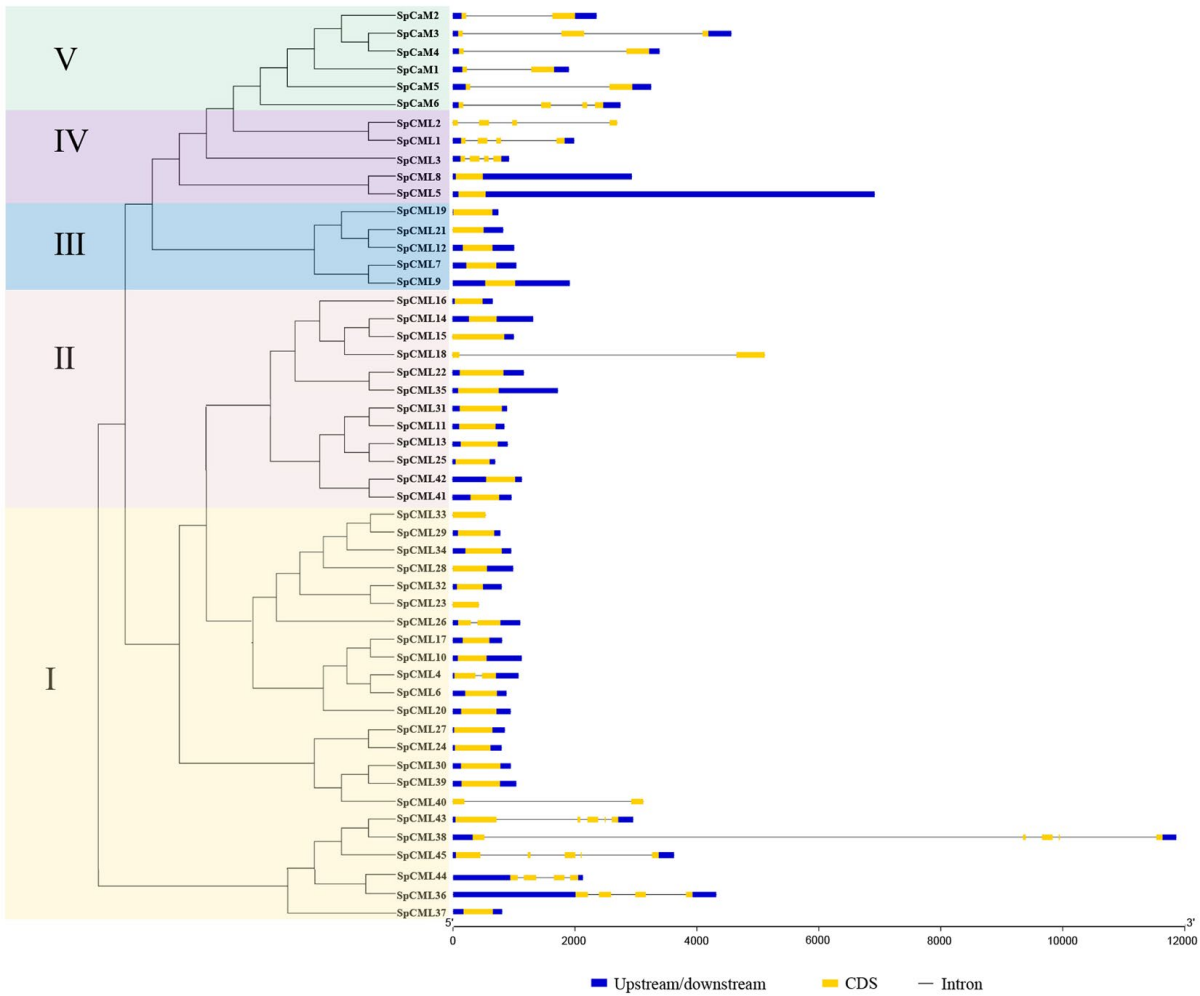
The genetic structure of SpCaM and SpCML family members. The phylogenetic tree was constructed using the full-length protein sequences of 6 SpCaM and 45 SpCML. Introns and exons of the *SpCaM* and *SpCML* genes were grouped according to the phylogenetic classification. Upstream/downstream, exons and introns were represented by blue boxes, yellow boxes, and the black lines respectively.

The exon-intron structures of the genes can provide significant evidence to support the phylogenetic relationships within a gene family^42^, and so genetic structure analysis of the *SpCaM* and *SpCML* were carried out using tools available with online website GSDS^43,44^ (Fig. 3). The analysis of the exons and introns of the *CaM* and *CML* genes enabled the genetic structure of these genes to be further understood. Five groups in the *SpCaM* and *SpCML* families were observed, which were consistent with the respective corresponding phylogenetic relationships depicted in Fig. 1. Fifteen genes in group I, all members of group II (except *SpCML18*), and all members of group III contained only one exon each. The *SpCML18* gene (group II) contained one intron and two exons, as did *SpCML4*, *SpCML26*, and *SpCML40* of group I. In group I, *SpCML36, SpCML38, SpCML43, SpCML44*, and *SpCML45* formed a small cluster containing 4-5 exons and 3-4 introns. Group IV was different from the other four groups and could be divided into two subgroups: one subgroup (*SpCML1-3*) contained four exons and three introns; the other had only one exon and no intron. All *SpCaM* genes belonged to group V, which involved 2-4 exons and 1-3 introns. Group I and IV genes possessed complex structures, suggesting that gene divergence occurred during evolution.

### Conserved motif analysis of the SpCaM and SpCML proteins

**Figure 4:**
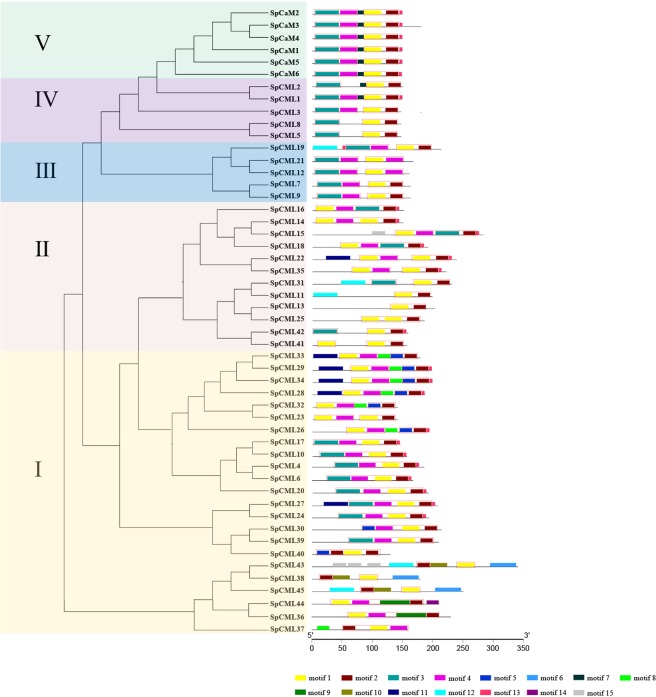
The conserved motifs of SpCaM and SpCML proteins. The phylogenetic tree was constructed using the full-length protein sequences of 6 SpCaM and 45 SpCML. The conserved motifs of SpCaM and SpCML proteins were grouped according to the phylogenetic classification. All motifs were identified by MEME. The motifs 1, 2, 3, 4, 5, and 9 were annotated as EF-hand domains. EF-hands were marked in red border rectangle.

To ascertain the feature sequences of the SpCaM and SpCML protein families, the program MEME^45^ was used to analyze the conserved motifs of the 51 genes based on their phylogenetic classifications, and 15 conservative motifs were identified in these proteins (Fig. 4, Table 2). The motifs 1, 2, 3, 4, 5 and 9 were annotated as EF-hand domains by the InterProScan, and the EF-hand domains in motifs 1 and 3 were more complete than those in motifs 5 and 9 (Table 2). As shown in Fig. 4, in addition to SpCML2, SpCML8, SpCML5, SpCML25, SpCML31, SpCML37, SpCML41, and SpCML42 lacked a EF-hand domain, and SpCML11, SpCML13, SpCML38, SpCML45, and SpCML43 lacked two EF-hand domain, the remaining SpCML and SpCaM all contained four EF-hand domain. The degenerate EF-hands in SpCML did not correspond to motif 6, 7, 8, 10, 11, 12, 13, 14, and 15.

**Table 2:** The amino acid sequence of conserved motifs of SpCaM and SpCML proteins.

Motif No.	Amino acid sequence^a^
motif1	LKEAFKVFDKDGBGYISAAELRHVLKNLG
motif2	CKEMIREVDVBGDGVINFEEF
motif3	LTDDQJSELKEAFSLFDKBGDGKITTEELGTVLRSLGQNP
motif4	EEELZDMINEVDABGBGFIDFEEFLNLMA
motif5	GYITPKELKSVLSRLGESQGI
motif6	EYDNFIECCLTVKGLTEKFKEKDTSYSGSATFTYDSFMLTILPF
motif7	RKMKDTDSEEE
motif8	IEEERBKESDLREAFNVFDQE
motif9	IGSPELEATFNTIVEAFLFLDKNGDGKLHKKDVLKALNDECPCEKSPSHV
motif10	QKALSSYNQSFGLRTVHLLMYLFTNTNAR
motif11	STAEKESFFSRLRNMFHLKKKEDEKKTTESATTTTTTSTST
motif12	MEPSNSLNRPNYKKFPHHTQPVPLLIHGASGFFFLYIIFDP
motif13	KVMMAR
motif14	WVGIDTDDEKGRLRATTTPQP
motif15	PPSWFGQKPPQSRSPAPPQPSPV

^a^The amino acid sequence of EF-hands are underline by black line.

The motif structures of the SpCML proteins in groups I and II showed diversity and complexity. All SpCML proteins from group I, except for SpCML40, SpCML43, SpCML38, and SpCML45, contained motifs 1, 2, and 4; motifs 5, 6, 8, 9, 10, and 14 occurred only in group I. Motifs 11 and 15 appeared only in groups I and II, and only in two genes, SpCML43 (group I) and SpCML15 (group II) contained motif 15. Five SpCML, including SpCML43 (group I), SpCML45 (group I), SpCML11 (group II), SpCML31 (group II), and SpCML19 (groupIII), harboured motif 12. Only SpCML19 in group III contained motif 13. In group IV, motifs 4, 7, and 13 occurred simultaneously in SpCML1. However, the structures of the SpCaM proteins were more regular in group V; these proteins contained motifs 7 and 13 in addition to the four EF-hand domains. Although some paralogous proteins contained distinct motif structures, such as SpCML23/32, SpCML30/39, SpCML38/43, and SpCML41/42, most paralogous proteins exhibited similar motif structures, including SpCML4/6, SpCML5/8, SpCML10/17, SpCML11/31, SpCML13/25, SpCML22/35, SpCML24/27, SpCML29/33, and SpCaM3/4.

Taken together, these results revealed that all identified proteins carried typical EF-hand domains, and each subgroup shared similar motif features. These results further support the phylogenetic classifications of the SpCaM and SpCML families.

### Cis-Element analysis of *SpCaM* and *SpCML* genes

To investigate the mechanisms of the stress-induced gene expression, the online database PlantCARE^46^ was used to analyze the cis-elements of the 2000 bp upstream sequences of the promoter regions for the *SpCaM* and *SpCML* gene coding sequences (Table 3). The results revealed that cis-acting elements associated with responses to phytohormones, such as abscisic acid (ABRE), salicylic acid (TCA-element and W-box), gibberellin (GARE-motif), methyl jasmonate (CGTCA-motif), ethylene (ERE) and auxin (TGA-element); adversity, such as anoxia stress (ARE), low temperature (LTR), light (Sp1 and I-box), drought (MBS), dehydration (DRE); and defense and stress-related elements (TC-rich repeats) occurred widely in the promoter regions of the *SpCaM*, and *SpCML.* 78.4% of the 51 genes contained ARE, while 72.5% contained ABRE, and  70.6% contained  CGTCA-motif and ERE. Other cis-elements (W-box, MBS, I-box, TC-rich repeats, TCA-element, TGA-element, LTR) accounted for a relatively small proportion of these genes, about 30 to 58%. In addition, there were three cis-elements that accounted for less than 18%, including the GARE-motif (17.6%), DRE (7.8%), and Sp1(2.0%). The fact that *SpCaM* and *SpCML* genes had the same or different cis-acting elements suggested that these genes may be simultaneously regulated in response to stress sometimes, or specifically regulated at other times when plants resist adverse external environments. These genes are involved in responses to different stresses.

**Table 3:** Cis-element analysis in the promoter regions of the *Solanum pennellii* SpCaM and SpCML proteins.

Gene family	Gene name	cis-acting element													
		ABRE	ARE	CGTCA-motif	DRE	ERE	GARE-motif	I-box	LTR	MBS	Sp1	TC-rich repeats	TCA-element	TGA-element	W-box
CaM	SpCaM1	+	+	+			+	+	+	+	+		+	+	+
	SpCaM2		+	+	+	+			+	+				+	
	SpCaM3	+	+	+						+					
	SpCaM4		+					+		+					+
	SpCaM5	+	+		+	+		+		+				+	
	SpCaM6		+	+		+							+		
CML	SpCML1	+	+	+		+		+	+			+		+	+
	SpCML2	+	+	+		+						+	+		+
	SpCML3	+				+			+						+
	SpCML4	+	+			+						+	+		
	SpCML5	+				+		+							
	SpCML6	+	+	+		+		+	+						
	SpCML7	+	+	+		+		+				+			
	SpCML8	+	+		+					+			+		
	SpCML9	+	+	+		+									
	SpCML10	+	+	+		+	+	+							+
	SpCML11	+		+		+						+			+
	SpCML12	+	+						+			+		+	+
	SpCML13	+	+	+		+				+		+			
	SpCML14	+	+					+					+		+
	SpCML15		+	+		+						+	+	+	+
	SpCML16	+	+	+					+	+			+		
	SpCML17	+	+			+				+					+
	SpCML18	+	+			+	+		+	+					+
	SpCML19		+	+			+	+	+	+			+		+
	SpCML20	+	+						+	+		+			+
	SpCML21	+	+	+			+			+			+		+
	SpCML22	+	+	+			+			+					+
	SpCML23		+	+		+				+					+
	SpCML24			+				+	+						+
	SpCML25		+	+			+	+				+			
	SpCML26	+	+	+		+			+	+		+	+	+	+
	SpCML27	+	+	+		+	+	+		+		+			
	SpCML28	+	+	+		+		+		+					
	SpCML29	+	+	+		+		+					+	+	+
	SpCML30	+				+			+			+			+
	SpCML31							+	+	+			+	+	+
	SpCML32	+	+	+		+				+				+	+
	SpCML33		+	+		+		+							+
	SpCML34		+	+				+		+		+	+	+	
	SpCML35	+	+	+		+				+		+		+	
	SpCML36	+		+		+		+	+	+				+	
	SpCML37	+		+		+	+						+		+
	SpCML38	+	+			+				+		+	+		+
	SpCML39		+	+		+				+		+			+
	SpCML40	+				+			+			+	+		+
	SpCML41	+		+		+								+	
	SpCML42	+		+		+							+		
	SpCML43	+	+	+		+		+						+	
	SpCML44		+	+		+			+						
	SpCML45		+	+	+	+		+		+		+		+	+
	Total	37	40	36	4	36	9	21	17	26	1	19	18	16	29

### Expression of *SpCaM* and *SpCML* genes in different tissues

**Figure 5:**
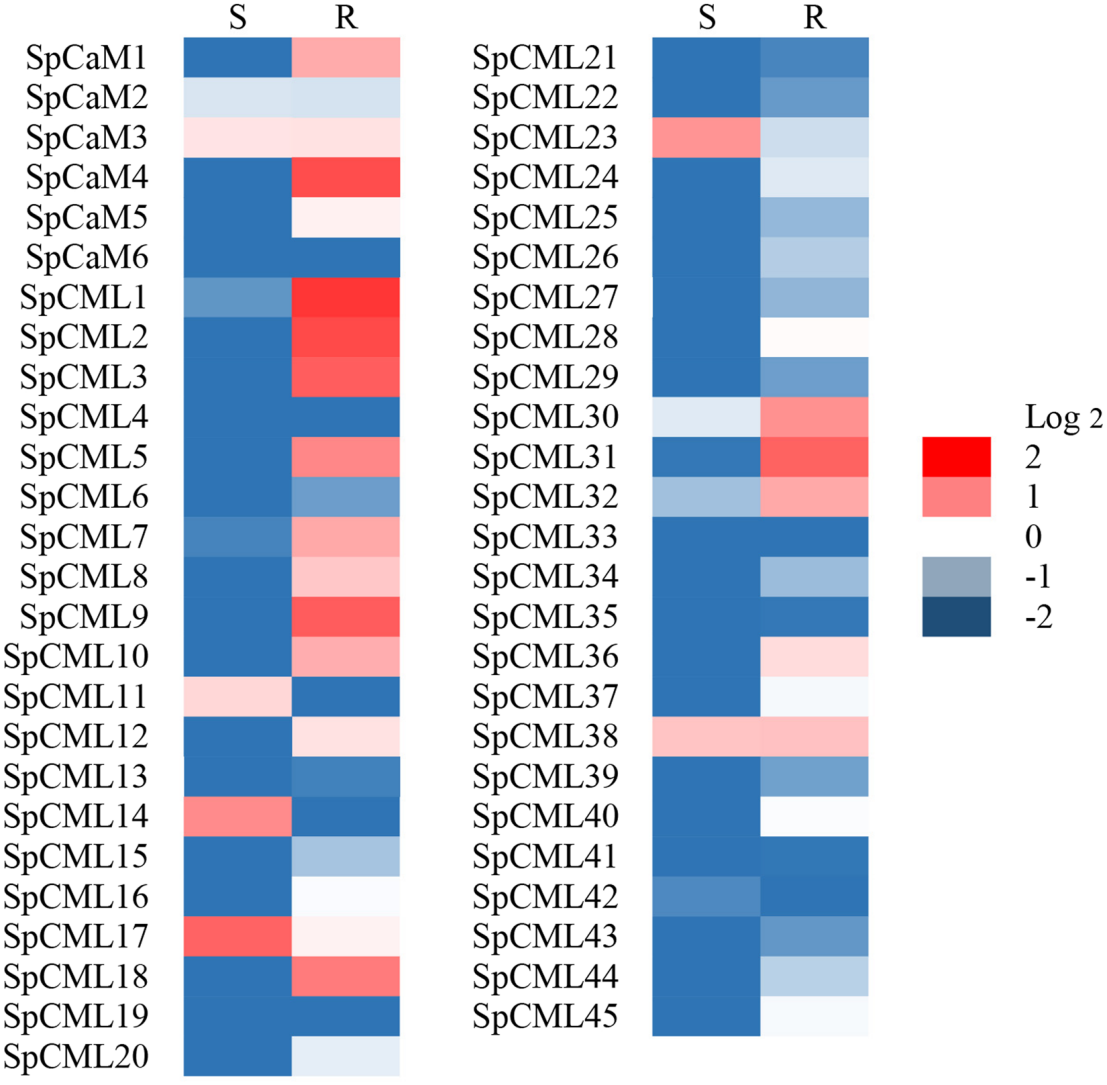
The expression heat map of the SpCaM and SpCML genes in stems (S) and roots (R) relative to leaves (Log2 = 0).

As shown in Fig. 5, transcripts of 51 genes were tested in all tissue samples, which revealed various expression levels of genes. Heat map displayed the expression level of the *SpCaM* and *SpCML* genes in stems (S) and roots (R) relative to leaves (Log2 = 0). One *SpCaM* and two *SpCML* genes—*SpCaM3*, *SpCML17*, and *SpCML38*—were upregulated in both stems and roots. *SpCML14, SpCML17*, and *SpCML23* showed high expression levels in stems. And *SpCaM1, SpCaM4, SpCML1, SpCML2, SpCML3, SpCML5, SpCML7,SpCML9, SpCML10, SpCML18, SpCML30, SpCML31*, and *SpCML32* were also highly expressed in roots. The tissue-based expression results indicated that *SpCaMs* and *SpCMLs* showed the specificity of gene function during plant growth and development.

### Expression of *SpCaM* and *SpCML* genes to abiotic stress and hormone treatments in different tissues

**Figure 6:**
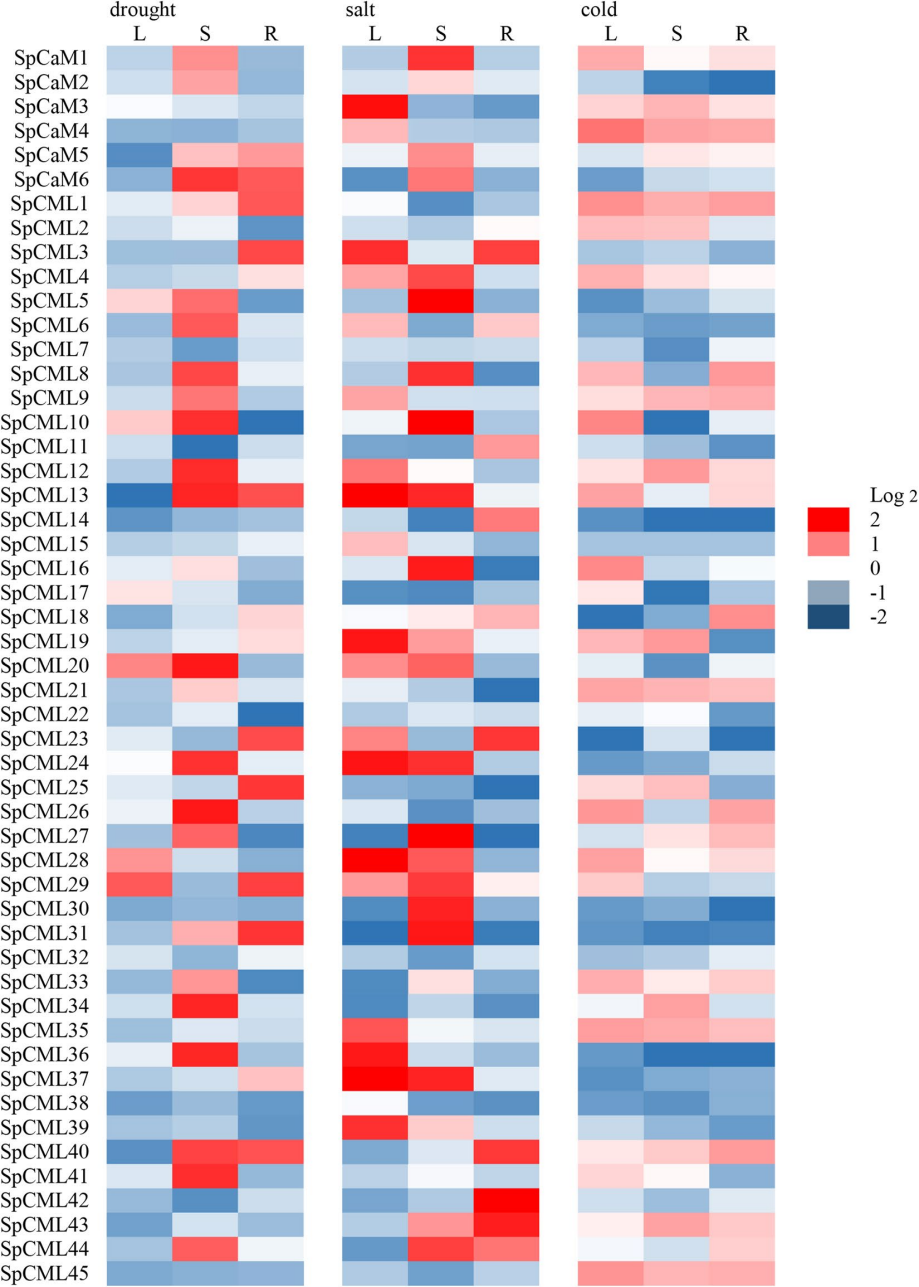
Expression profiles of the *SpCaM* and *SpCML* genes under abiotic stress in different tissues. S: stems, R: roots, L: leaves.

The results showed that all *SpCaM* and *SpCML* genes were expressed under cold, drought, and salt stress, but exhibited disparate relative expression levels in different tissues following stress treatments (Fig. 6). In leaves, 6, 19, and 25 of the 51 genes showed high expression (Log2 > 0) under drought, salt, and cold stress, respectively. In stems, 25, 25, and 21 genes were upregulated (Log2 > 0) under drought, salt, and cold stress, respectively. In roots, 14, 12, and 21 genes were induced (Log2 > 0) under drought, salt, and cold stress, respectively. Remarkably, in leaves, drought and cold stress induced strong expression of only one gene each (Log2 > 1), namely *SpCML29* and *SpCaM4*, respectively, whereas 11 genes (*SpCaM3, SpCML3, SpCML12, SpCML13, SpCML19, SpCML24, SpCML28, SpCML35, SpCML36, SpCML37*, and *SpCML39*) were strongly expressed (Log2 > 1) in leaves under salt stress. Conversely, in stems and roots, no genes were obviously upregulated (Log2 > 1) under cold stress. However, 17 genes showed strong expression (Log2 > 1) under drought and salt stress respectively, in stems. Nine and seven genes were also overexpressed (Log2 > 1) under drought and salt stress, respectively, in roots. On the whole, the total number of strongly upregulated (Log2 > 1) genes were higher in stems than in leaves and roots. In addition, under cold stress, 14 genes (*SpCaM1, SpCaM3, SpCaM4, SpCML1, SpCML4, SpCML9, SpCML12, SpCML21, SpCML28, SpCML33, SpCML35, SpCML40, SpCML43*, and *SpCML45*) were upregulated simultaneously (Log2 > 0) in leaves, stems, and roots. But, this phenomenon did not appear under salt and drought stress.

**Figure 7:**
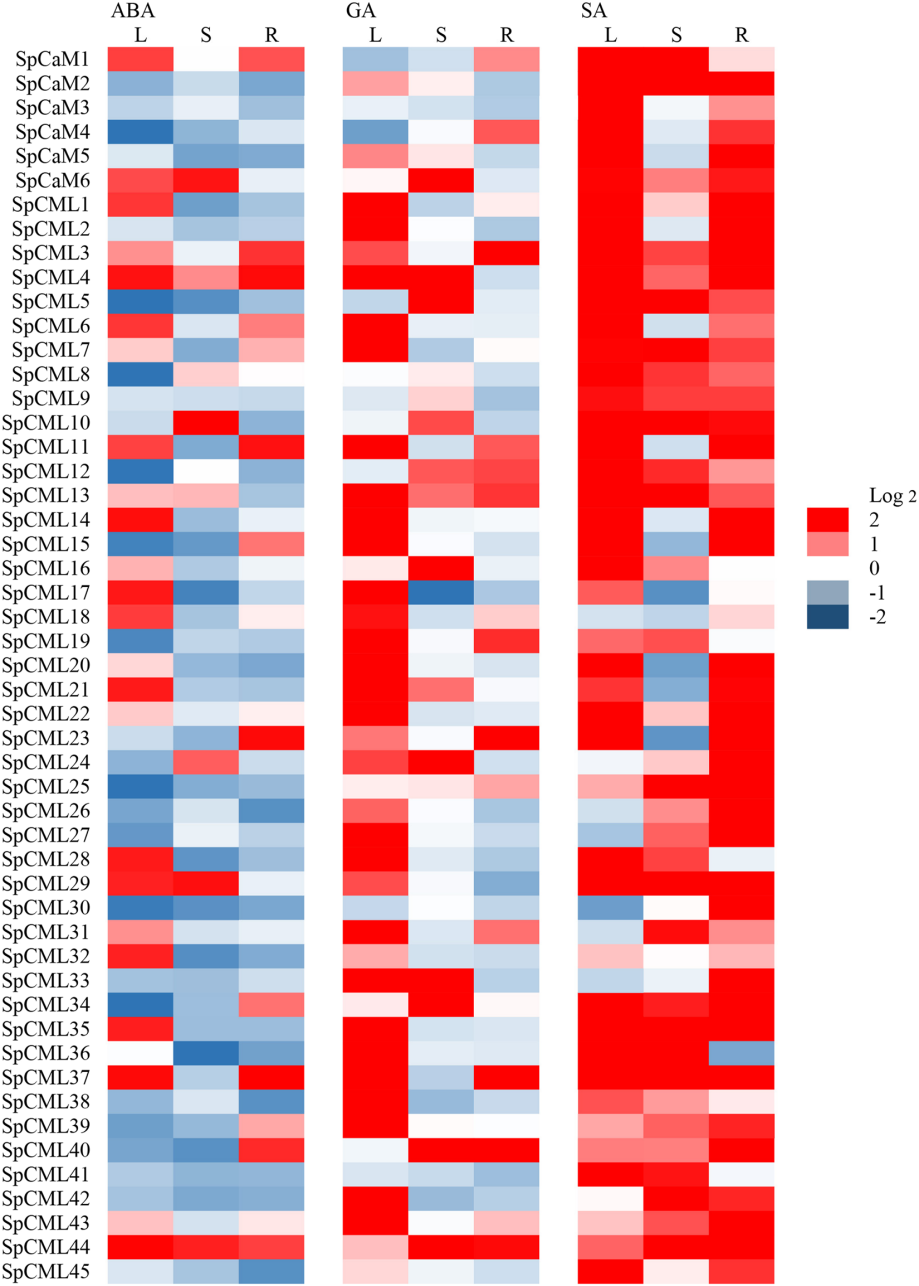
Expression profiles of the *SpCaM* and *SpCML* genes under hormone treatments in different tissues. S: stems, R: roots, L: leaves.

The expression levels of *SpCaM* and *SpCML* genes under ABA, GA, and SA treatments are depicted in Fig. 7. ABA treatment upregulated (Log2 > 0) 24, 8, and 16 genes in leaves, stems, and roots, respectively. GA treatment induced (Log2 > 0) 40, 19, and 18 genes in leaves, stems, and roots, respectively. SA treatment upregulated (Log2 > 0) 44, 37, and 46 genes in leaves, stems, and roots, respectively. These results indicated that a higher number of *SpCaM* and *SpCML* genes were upregulated in response to SA than in response to ABA and GA. Notably, in leaves, 16, 31, and 39 genes showed strong expression (Log2 > 1) upon ABA, GA, and SA treatments. In stems, 5, 13, and 28 genes were strongly induced (Log2 > 1) by ABA, GA, and SA treatments. In roots, 11, 11, and 38 genes were also overexpressed (Log2 > 1) by ABA, GA, and SA treatments. Thus, the total number of strongly upregulated genes was higher in leaves than in stems and roots. The analysis also showed that 27 genes were induced simultaneously (Log2 > 0) in leaves, stems, and roots by SA treatment, while 2 (*SpCML4* and *SpCML44*) and 4 genes (*SpCML13, SpCML25, SpCML34*, and *SpCML44*) were upregulated simultaneously in leaves, stems, and roots, respectively, by ABA and GA treatments. These results suggested that *SpCaM* and *SpCML* genes may be associated with plant resistance to abiotic stress and regulatory hormones and that different members may play different roles in response to different stimuli.

## Discussion

Ca^2+^, as a multifunctional signaling molecule, is at the core of complex antistress signaling pathways in response to adverse environmental conditions^47^. Calmodulin (CaM) is the main calcium sensor in all eukaryotes and can sense changes in the concentration of Ca^2+^. Change of intracytoplasmic free Ca^2+^ level is the earliest response of cells to various abiotic and biological stresses^5^. Recent studies have found that CaM and CMLs are key components of stress signal transduction. For instance, heat shock proteins (HSPs) induced by high temperatures are regulated by heat shock transcription factors (HSFs). HSFs are activated by phosphorylation of protein kinases. Compared with wild type, the activity of HSFs in the AtCaM3 mutant was decreased, which inhibited the biosynthesis of HSPs and reduced the heat resistance of plants^48^. The overexpression of the soybean GmCaM4 gene activates the pathogenesis-related (PR) gene and accumulates jasmonic acid (JA), which increases soybean resistance to the oomycete Phytophthora sojae, Alternaria tenuissima and Phomopsis longicolla. However, the silencing of the GmCaM4 gene significantly inhibited the expression of the PR gene^49^. MYB2 (CaM binding transcription factor) contains a Ca^2+^-dependent CaM binding domain and regulates the expression of salt and dehydration response genes in *Arabidopsis*^50^. It has been confirmed that the interaction of GmCaM4 and MYB2 regulates the expression of salt-responsive genes and improves tolerance to high-salt environments^49^. AtCML42 mutant increases the expression of JA responsive gene, thus enhancing the plant defense against herbivorous insects. In addition, JA-induced Ca^2+^ elevation and root growth inhibition are more pronounced in AtCML42 mutants. The above results indicate that AtCML42 is an important component connecting Ca^2+^ and JA signals, and plays a negative regulatory role. AtCML42 is also involved in abiotic stress responses. AtCML42 mutant decreases resistance to ultraviolet radiation B (UV-B) and accumulates abscisic acid content under drought stress^51^. The difference is that the accumulation of JA in ATCML37 mutants is significantly reduced, which indicated ATCML37 plays an active regulatory role in Ca^2+^ signaling pathway^52^. These data suggest that CaM and CML mediate multiple defense signaling pathways. *Solanum pennellii* possesses excellent resistance to stress, which is an important germplasm resource to cultivate high-quality tomato^53^. However, the structural characteristics of CaM and CML genes and their responses to various stresses have not been systematically studied in *Solanum pennellii*. In this study, we identified and systematically analyzed the two families.

We identified 6 CaM and 45 CML from the *Solanum pennellii* genome sequence. This is roughly consistent with the number of genes identified in other plant species previously reported, such as *Arabidopsis*^9^, *Oryza sativa*^13^, *Gossypium raimondii*^11^, and *Nicotiana Benthamiana*^8^ (Table 4). The results showed that there were differences in the number of genes in CaM and CML families (Table 4). The differences in sizes of genes in these families may be due to their ploidy levels and their involvement in different vital cellular processes. In principle, adding or evolving more genes or genomes is the inevitable result of and the correct direction for plant evolution. This phenomenon may occur because ecological strategies of different plants to cope with different environments are related to the adaptation and expansion of gene families^54–56^. Over the course of evolution, variations in gene family size are mainly caused by natural variation in different species and their adaptation to complex growth environments^56,57^.

**Table 4:** The number of CaM and CML in different species.

Species	Protein type	
	CaM	CML
*Solanum pennellii*	6	45
*Arabidopsis*	6	50
Rice	5	32
*Gossypium raimondii*	6	30
*Nicotiana Benthamiana*	7	55

As a result of the abundant selective splicing of genes and the post-translational modification of proteins, the functional and chemical complexity of proteins is enhanced. The post-translational modification event myristoylation has extremely diverse biological functions associated with signal transduction, protein transport, protein localization, extracellular communication, and protein regulation and metabolism. The analysis showed that SpCaM was without myristoylation sites (Table 1). Palmitoylation and myristoylation are sometimes interrelated and interdependent, and the absence of myristoylation may lead to the disappearance of palmitylation^58^.

Phylogenetic trees were constructed to understand the evolution of SpCaM and SpCML (Fig. 1). The SpCaM and SpCML were classified into five groups (I, II, III, IV, and V). The results showed that CML groups (I, II, III, IV) dominated the phylogenetic tree. SpCaM and SpCML evolved together from their common ancestors, and these SpCML evolved before SpCaM. This is why there are more *SpCML* genes than *SpCaM* genes in the genome, and *SpCML* genes were diversified more. Location analysis of these 51 genes on the chromosome revealed that these genes were not evenly distributed on the chromosome (Fig. 2). Chromosomes 3 had the most genes (8 genes), followed by chromosomes 1, 4, and 11 (7 genes each) and chromosome 2 (6 genes). Chromosomes 1, 3, 10, 11, and 12 contained both *CaM* and *CML* genes (Table 5).

**Table 5:** The number of genes on the chromosomes of *Solanum pennellii*.

Gene name	Chrom 1	Chrom 2	Chrom 3	Chrom 4	Chrom 5	Chrom 6	Chrom 7	Chrom 8	Chrom 9	Chrom 10	Chrom 11	Chrom 12	Total
CaM	1	0	1	0	0	0	0	0	0	2	1	1	6
CML	6	6	7	7	1	4	1	1	2	3	6	1	45
Total	7	6	8	7	1	4	1	1	2	5	7	2	51

The conserved motif and gene structure analyses of *SpCaM* and *SpCML* genes showed that each group shared similar exon-intron structures and motifs, which provided further evidence for their classification (Figs. 3 and 4). Gene structure analysis showed that most *SpCML* genes lacked introns, while *SpCaM* contained only one long intron (Fig. 3); these findings were in accordance with findings on the exon-intron structure of *CaM* and *CML* genes in *Arabidopsis*^9^, *Nicotiana benthamiana*^8^, *Brassica rapa* L.^16^, and *Solanum tuberosum*^8^. However, some *SpCML* genes contained 1, 3, or 4 introns. At present, studies on the evolution of introns have found that intron loss is more likely to occur than intron gain during evolution^59^. Based on these insights, it can be hypothesized that the majority of *SpCML* without introns are older than *SpCaM*. The few *SpCML* genes with introns possibly evolved from their closest *SpCaM*. This explains why group IV *SpCML* and group V *SpCaM* are the closest in the evolutionary tree (Fig. 1). The conserved motif is also a key index to evaluate protein function^60^. The exon-intron distribution analysis reflected the conservatism and functional differences among different proteins. Conserved motif analysis suggested all SpCaM proteins contain 4 EF-hand type calcium-binding domains, and all SpCML contain at least 1 EF-hand type calcium-binding domain (Fig. 4).

qRT-PCR analysis of *SpCaM* and *SpCML* indicated that the expression levels of *SpCaM* and *SpCML* genes were affected in *Solanum pennellii* under abiotic stress and hormone treatments. The expression profiles of *SpCaM* and *SpCML* genes in different tissues showed different expression levels of *SpCaM* and *SpCML* genes (Fig. 5). The *SpCaM4*, *SpCML1, SpCML2*, *SpCML3*, *SpCML9*, *SpCML18*, and *SpCML31* showed significantly higher expression level (Log2 > 1) in roots than in other tissues, while expression level of *SpCML17* in stems was significantly higher (Log2 > 1) than in other tissues, suggesting that different *SpCaM* and *SpCML* gene members have distinct expression levels in various tissues. The diversified expression of these *SpCaM* and *SpCML* genes revealed that they might play a significant role in different plant tissues^61^.

The expression levels of *SpCaM* and *SpCML* genes under abiotic stress and hormone treatments in different tissues indicated that the expression of *SpCaM* and *SpCML* genes were affected (Figs. 6 and 7). Under cold stress, the expression of SpCaM4 in leaves was significantly increased (Log2 > 1), while down-regulation of S*pCaM4* expression was found under ABA treatment, revealing that *SpCaM4* may be involved in Ca^2+^ transport under cold stress. The results are not entirely consistent with previous studies. Delk *et al*. found *Arabidopsis CML24* was expressed in all major organs and upregulated under cold stress and ABA treatment^62^. It is has been reported that *AtCML9* was induced under salt stress and ABA treatment, and involved in salt stress tolerance by affecting ABA-mediated pathways^63^. In *Solanum pennellii*, the expression levels of 11 genes were obviously upregulated (Log2 > 1) under salt stress, ABA and GA treatments, including 3 (*SpCML28, SpCML35* and *SpCML37*), 4 (*SpCaM6*, *SpCML10*, *SpCML24* and *SpCML44*) and 4 (*SpCML3*, *SpCML23*, *SpCML40* and *SpCML44*) genes in leaves, stems and roots. These ten genes might participate in salt stress via ABA and GA-mediated pathway.

Under drought, salt, and ABA treatments,   4 (*SpCaM6*, *SpCML1*0, *SpCML24*  and *SpCML44*) and 3 (*SpCML3, SpCML23 and SpCML40*) genes in stems and roots showed strong expression (Log2 > 1) (Figs. 6 and 7). The results are consistent with the study by Xu *et al*. who reported *OsMSR2* (*Oryza sativa* l. multi-stress response gene 2), a novel *CML* gene, was strongly upregulated under drought and salt stress in different tissues at different stages of development, and enhanced tolerance to salt and drought via ABA-mediated pathway in rice^61^.

Conversely, *Arabidopsis AtCML37*, *AtCML38*, and *AtCML39* showed greater sensitivity to drought and salt than to ABA and SA, suggesting that these proteins may act as Ca^2+^ transducers in signaling pathways independent of ABA and SA^64^. OsCML4 confers drought tolerance through ROS-scavenging in an ABA independent manner in rice^65^. This phenomenon also exists in this study. The expression of the *SpCML20* gene in stems was significantly induced (Log2 > 1) under drought and salt than under ABA and SA  (Figs. 6 and 7). These results suggested that SpCaM and *SpCML* genes have diverse functions in different tissues in response to different stimuli, and may play a role as stress response genes to improve stress tolerance.

In this study, a total of 6 *CaM* and 45 *CML* genes were identified in the *Solanum pennellii* genome. These 51 genes were unevenly located on 12 chromosomes. SpCaM and SpCML were classified into five groups via phylogenetic analysis. Further analysis of their conserved motifs and gene structure revealed their evolutionary relationship, wherein it was suggested that SpCML evolved earlier than SpCaM. Analysis of cis-acting elements of these genes implied that they play crucial roles in response to multiple signaling pathways related to stress resistance. This study provides important insights into the evolution and function of *Solanum pennellii* genes, which lays a good foundation for the genetic improvement of stress-resistant tomato cultivars.

## Materials and Methods

### Identification of SpCaM and SpCML

All CaM and CML protein sequences of *Arabidopsis* and rice were obtained from the TAIR database (http://www.arabidopsis.org/) and rice Database (http://rice.plantbiology.msu.edu/), respectively. The whole protein and nucleotide sequences of *Solanum pennellii*^33^ were obtained from NCBI (https://www.ncbi.nlm.nih.gov/genome/).

### Phylogenetic analysis and chromosomal localization

CaM and CML protein sequences of *Arabidopsis* (6 and 50, respectively)^12^ and rice (5 and 32, respectively)^13^ and SpCaM and SpCML protein sequences of *Solanum pennellii* were aligned by the MUSCLE program of MEGA6.0^40^, with default settings. Then, phylogenetic trees were constructed using the neighbor-joining method of MEGA6.0, in which bootstrap value was set to 1000. The chromosomal location information of 51 genes of *Solanum pennellii* was obtained from the NCBI database. The online MapGene2Chrom program was used to map their chromosomal locations (http://mg2c.iask.in/mg2c_v2.0/)^41^.

### Sequence analysis

Physicochemical parameters of SpCaM and SpCML proteins, including theoretical isoelectric point (pi), molecular weight, amino acid sequence length (AA), and the N-terminal myristoylation were predicted using the ExPASy proteomics server (http://web.expasy.org/myristoylator/), with default settings^38^. The ScanProsite tool of ExPASy was used to retrieve the EF-hand domain, and calcium-binding region. The subcellular localization of proteins was predicted using the Wolf PSORT (http://www.genscript.com/psort/wolf_psort.html) program^39^. The structure of these genes was analyzed using tools available with online website GSDS (http://gsds.cbi.pku.edu.cn/)^43,44^. Genomic DNA sequences of SpCaM and SpCML were downloaded from the NCBI database.

### Conserved motif analysis

The MEME suite (http://alternate.meme-suite.org/tools/meme) was used to identify 15 conserved motifs. These conserved motifs were further annotated with InterProScan^45^.

### Cis-acting element analysis

The starting site of SpCaM and SpCML nucleotide sequences on chromosomes were searched in NCBI. The upstream 2000 bp sequences of *SpCaM* and *SpCML* genes, as the promoter region, were obtained using the same method, and then the database PlantCARE was used to analyze the cis-acting elements in the promoter region (http://bioinformatics.psb.ugent.be/webtools/plantcare/html/)^46^.

### Plant materials

In this study, wild cultivar *Solanum pennellii* LA0716 was used. The seeds were placed on moist filter paper in a petri dish, then the petri dish was transferred to a constant temperature incubator at 27 °C without light for 3 days. The germinated seeds were transplanted into 1/2 full nutrient solution^66^. All plants were grown in a 26 °C/19 °C (day/night) greenhouse at approximately 70% relative humidity and incubated for 30 days.

### Abiotic stress and hormone treatments

Thirty-day-old seedlings were used to explore the responses of the plant to abiotic stress and hormone treatments. For cold stress, salt stress and drought stress, the seedlings were respectively placed in 1/2 full nutrient solution at 4 °C, with 100 mmol/L NaCl, and with 10% polyethylene glycol (PEG) 6000. Drought stress was simulated by decreasing osmotic potential. For the hormone treatments, the seedlings were respectively grown in 1/2 full nutrient solution with 150 µmol /L gibberellic acid (GA), with 100 mmol /L abscisic acid (ABA), and with 100 µmol/L salicylic acid (SA). The seedlings were collected at 1 h after treatments. All the treatments collected three biological samples, which were immediately frozen in liquid nitrogen and stored at −80 °C for further analysis.

### RNA extraction and qRT-PCR assays

TRIzol reagent (Tianmo biotech, Beijing, China) was used to extract total RNA from the roots, stems, and leaves according to the manufacturer’s instructions. Then, DNase I treatment was used to removing genomic DNA contamination from total RNA. Two micrograms of total RNA were used for the first-strand cDNA synthesis using the 5X All-In-One RT MasterMix (with AccuRT Genomic DNA Removal Kit) (Applied Biological Materials, Zhenjiang, China). For qRT-PCR analysis, the reactions were performed using the Bestar® Sybr Green qPCR Master Mix (DBI, Shanghai, China) in an ABI7500 qRT-PCR system according to the manufacturer’s instructions The primers used for qRT-PCR analysis are listed in Table 6. For all analyses, actin was used as an internal control. Three technological replicates of each sample were assayed. The relative quantification of specific mRNA levels was calculated from the cycle threshold (Ct) using the 2^−ΔΔCt^ method^67^.

**Table 6:** Primers used in qRT-PCR analysis.

Gene Name	Forward primer	Reverse primer	Gene Name	Forward primer	Rever Se primer
SpCaM 1	GGATCAAAATGGCTTCATCTCC	CCATCAACATCAGCTTCCCTAA	SpCML21	CTACATTACCGCCGCTGAAC	CGGAGACGTTATTTAATCCGAGA
SpCaM 2	ATGGCAGATCAGCTCACCG	CCAACGACCTCATCACAGTCC	SpCML22	ATGAATGAAGAAGAAGTTGCTAA	TTACAATGATCCAATTTCTTACC
SpCaM 3	AGAGGTTGATGAGATGATTCGTG	CTTTTCTTCCATTGCTCTGTGA	SpCML23	GTAGAAGATGGAGGGACGAAAGA	TCTTCTCCCCTAACCTATGAAGC
SpCaM 4	ATGGCGGATCAGCTTACAGA	GATTTTGTCCCAGCGAACG	SpCML24	GTAAAGCAAACGCCAAGCAC	TCATCGCCGATTTGTATTCC
SpCaM 5	AACTTGGAACTGTAATGCGGTCA	CCCATTTCCATCAGCATCAAC	SpCML25	TTCTTGGATTTATCGGTGTTCG	AAGATATGCTCGATGAGCTTGAC
SpCaM 6	AGGATGGCGATGGCTGTATT	TGTCCTTCATCTTCCGTGCC	SpCML26	CAAAGTCTTCAAATTCAAGCCAA	TACCATCGTTATCGGTGTCAAA
SpCML1	TCCAACAGAGGAAGAACTGCC	GCTTGTCAAACACCTTGAAAGC	SpCML27	GGGGAAGAAGAACCTCCTCAC	AGGCACTCTGCAAATCCGTAG
SpCML2	AAGGTCATTGGATGAAAATCCA	GTTCTTCCTCTAAATCCGTCTCC	SpCML28	AGTAGTAGCGAAAATAGCGATCAA	ATCCTCTAACCCTAACATCCCAT
SpCML3	AAAATCCAACAAGGGAAGAGC	CCGTTACATTGTCCTTGAGCT	SpCML29	TAGTTTCTGTTTCCATGGCTGAA	CACTCTCATGTTCCTTCTTAGGAGT
SpCML4	ACGATAATCCAATGCCTGTGAA	GGACCCTAATTCCGATGAACATA	SpCML30	TACGAGCGTGTTACCTACTTTATCC	TCCTCTTCACTCGGTGGAGATT
SpCML5	CATAATCGCCGAGGAGAAAC	CGAGGACCTTAAAGGCATCA	SpCML31	TCCAGTTCCCCTGCTAATTCAT	GCCATAACCATTTCCACCTCTT
SpCML6	AGAGGAGCTACAACAAGTATTCAAC	CTCTGTTACAGCGGTTCCTAAAC	SpCML32	AAAGGCTGCTGTTGAGCATAAT	CACCCTTCCATTTCATACATCTTG
SpCML7	GATAGGGACGGTAATGGTGTGA	GAAGTCATCGCCTGAGCAAAC	SpCML33	ACAACAGCAACAACAACCACCA	CCCATCTCCGTTCTCATCAAAG
SpCML8	CATTTGATCGCCAATTACGC	GAAATCTTCATACCGGATCTTACC	SpCML34	GGAGATGGAAAGGTGTCACCTG	TAAAATCCTCAATGCCCAATAAT
SpCML9	TTTGATAGGGATGGAAATGGTTA	CCGAAGAAATCGCCTGAGTAA	SpCML35	CTCTCTGTTAAACCTCAAACCCTAA	ATCTCCATCTTCCATAAAAATCCC
SpCML10	AACTCCCAGCGACTCCAAAC	GACGTCTTACTGCCAAGTGCA	SpCML36	CTCTAGTCCCAAGGCTTCTATCC	GCTCCGTTGCTGTCTTCATC
SpCML11	CCGTTAGTACCGTTCCTGTTCC	CACATTATGGTGGGATGTTGTTG	SpCML37	AGTGTTTTGCATTCTGCCGG	GTCATCATCGTACCAATCATATCGT
SpCML12	CGGGCGATCAAATCCATACA	GCAGCCGTTATATAACCGTTACC	SpCML38	GTCAAGGGACTGACGGAGAAAT	AGCAATGAGGAAAGGCAGAATA
SpCML13	TTCATCATTGTGCGAAACGC	CTCGAAACCTCTGCATACTCTGTT	SpCML39	GCAGAGTCGGAACTGAACCA	GGTCCAAACGCTGAGCTAATAG
SpCML14	AATGGAAGAGCGAGATGAGGAG	GACCAAGTGATGCCAAGACAGA	SpCML40	TGGCGATGGACGTATTGATATG	CGGAATGCCTCCGTGAGAT
SpCML15	ATGTGAATGGAGACGGTTGC	AGCCCTAACGATGCGAGAAC	SpCML41	TTGGTAAAACAAGCCTAGACTTCA	CAGCTCCTCACAACATATGAATCC
SpCML16	CAATGAACGAGCTAAATGATTCG	GTTGAATGCCTCTTGCATGTCTT	SpCML42	AGATGATCTTGTTGAAGCATTCAAG	CTTACAATCACTTCCTTCTTTCTCA
SpCML17	AGCGAATGATGCTAGAGGTTGA	CTCTCCAATGCCTTTCATAACG	SpCML43	CCACTCACAACCGTCTGCTC	CATCAATTAACCCACTCCCATC
SpCML18	TTTTGTCATCGTTGGGTTTGAA	CCACCACGTTTCATCATTTGTCT	SpCML44	AGGGGTTGAAGGAGATAAAAGAC	TTCATCCACATCACAAGAGTCG
SpCML19	TTCTAATGGAAATGGGTCTGTGG	TCTCGATACGACAATGGCTGAC	SpCML45	ACCCAAATGAGCCACCAATAG	AAGAACCAGGTCCATAACCAGC
SpCML20	GATTGATACAAATGGGGATGGA	CTGCTAATTCTTCAGCAGCTTTC	Actin	ATTCAGCCCCTTGTTTGTGAC	CATAGGCATCCTTCTGTCCCAT
